# Detecting differentially expressed genes for syndromes by considering change in mean and dispersion simultaneously

**DOI:** 10.1186/s12859-018-2354-4

**Published:** 2018-09-20

**Authors:** Chenchen Ma, Tieming Ji

**Affiliations:** Department of Statistics, University of Missouri at Columbia, Columbia, 65211 MO USA

**Keywords:** Empirical Bayes, Gene expression, Testing mean and variance, Syndrome

## Abstract

**Background:**

Using next-generation sequencing technology to measure gene expression, an empirically intriguing question concerns the identification of differentially expressed genes across treatment groups. Existing methods aim to identify genes whose mean expressions differ among treatment groups by assuming equal dispersion across all groups. For syndromes, however, various combinations of gene expression alterations can result in the same disease, leading to greater heteroscedasticity in the biological replicates in the disease group compared to the normal group. Traditional methods that only consider changes in the mean will fail to fully analyze gene expression in such a scenario. In addition, sequencing technology is relatively expensive; most labs can only afford a few replicates per treatment group, which poses further challenges to reliably estimating the mean and dispersion under each treatment condition.

**Results:**

We designed an empirical Bayes method and a pooled permutation test to simultaneously consider the change in mean and dispersion across treatment groups. We further computed confidence intervals based on Bayes estimates to identify differentially expressed genes that are unique to each disease sample as well as those that are common across all disease samples. We illustrated our method by applying it to gene expression data from a large offspring syndrome experiment, which motivated this study. We compared our method to competing approaches through simulation studies that mimicked the real datasets to demonstrate the effectiveness of our proposed method.

**Conclusions:**

We will show that, compared to popular methods that only aim to find the difference in the mean, our method can capture greater variation in the disease group to effectively identify differentially expressed genes for syndromes.

## Background

Differentially expressed (DE) genes often refer to genes whose mean expressions differ across treatment groups, such as normal versus disease groups. Significant DE genes in the genome are considered to be related with the disease of interest. Thus, reliable detection of DE genes is helpful for understanding the underlying mechanism of disease occurrence.

High-throughput technologies, such as microarray and next-genration sequencing (NGS) technology measure gene expression levels simultaneously for tens of thousands of genes in the whole genome. Compared to microarrays, NGS technology enjoys several advantages. NGS experiments measure the number of reads from a gene, which is closer to the natural measurement of RNA abundance than the fluorescence measurement from microarrays. Moreover, NGS provides expression measurements of similar transcripts that would be difficult to separately measure with microarrays due to cross-hybridization. NGS experiments also provide information of sequence variation, such as alternative splicing, allele specific expression, single nucleotide polymorphisms and so on [1, 2]. However, NGS is relatively expensive; hence, most biology labs can only afford three or four replicates per treatment group. With so few replicates, it is extremely challenging to accurately estimate expression means and error variances that are crucial to DE gene identification. Auer and Doerge [3] proposed a two-stage Poisson model (TSPM) that assumed NGS read counts for each gene following either a Poisson distribution or Poisson with overdispersion. Robinson and Smyth [4] proposed modeling NGS read count data as random variables following a negative binomial (NB) distribution. To improve estimation of the mean and dispersion parameters in the negative binomial distribution for each gene, [4] also assumed that gene dispersions, although they may vary across genes, were a sample from a common prior distribution. Thus, observations from tens of thousands of genes could be pooled to accurately estimate the common hyper priors and improve estimates of each individual gene dispersion. This method has been implemented in the edgeR package and is regarded as one of the most popular and effective methods for detecting DE genes. Similar to the idea in [4], [5] also used an NB model to borrow information across genes. They made an additional assumption of a locally linear relationship between variance and the mean expression levels. Their method has been implemented in the DESeq package. Hardcastle and Kelly [6] also adopted the NB model, but unlike the other two methods, they used the Bayes factor approach in hypothesis testing and ranked genes based on posterior probabilities. Their method was implemented in the baySeq package. Several simulation studies and real data analyses have found the edgeR, DESeq, and baySeq methods, which borrow information across genes, can greatly improve the power of DE gene detection over the naive generalized linear model without sharing information. Other developments include the Cuffdiff method [7], the NOISeq method [8], the BBSeq method [9], the BAGE method [10], the QuasiSeq method [11], the ShrinkBayes method [12], and the DESeq2 method [13]. These new developments share similar ideas with edgeR, DESeq, and baySeq but are expanded to include more specific situations. For example, the Cuffdiff method can detect DE genes with alternative splicings, the BAGE method can analyze data from multiple experiments simultaneously, and the QuasiSeq method uses a quasi-likelihood for simpler computation and better estimates of false discovery rate (FDR) control.

**Fig. 1:**
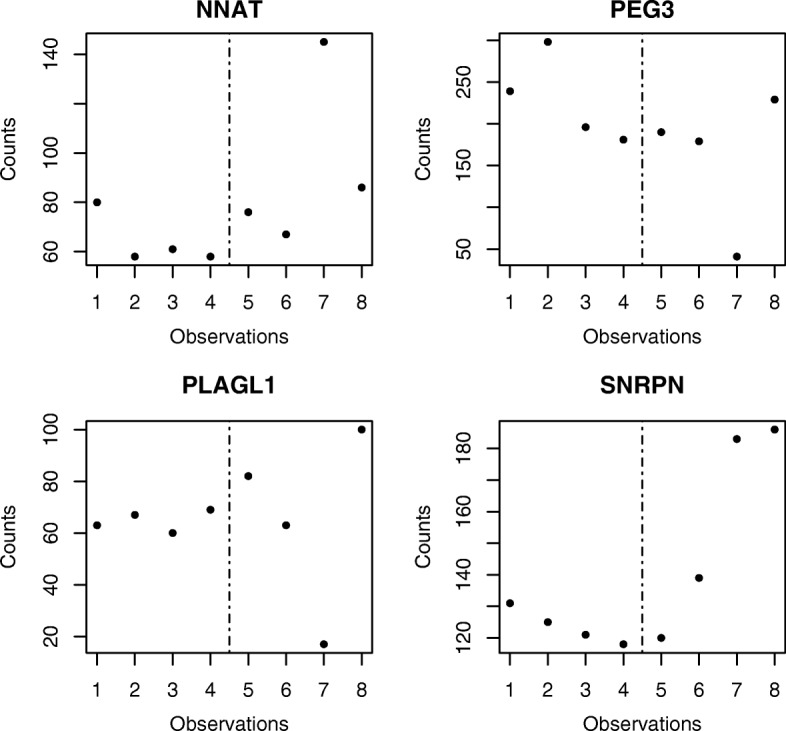
Four example genes. Normalized counts for example genes. The first 4 counts for each genes are from normal group, and the last 4 are from LOS group

Although previous studies have realized great advances, they all assume that mean expressions within one treatment group are the same among biological replicates. Yet this statistical assumption does not hold for certain disease groups. For example, syndromes include a group of various symptoms that co-occur to characterize a disease. Afflicted individuals exhibit different combinations of symptoms that manifest from the same disease. Thus, for DE genes related to the syndrome of interest, only some of the replicates in the disease group show differential expression. For instance, [14] studied large offspring syndrome (LOS). Figure 1 shows four genes – gene NNAT, PEG3, PLAGL1, and SNRPN – related to the occurrence of LOS [14, 15]. For each gene, the first four observations are from the control group, and the last four observations are from the LOS group. Specifically, for gene NNAT, replicate 3 in the LOS group expressed substantially differently, whereas LOS replicates 1, 2, and 4 expressed similarly to the normal group. For the other three example genes, it also happened that only some of the disease replicates showed differential expression. Using Sanger sequencing, genotyping results confirmed loss or gain of imprinting at these four gene loci for one or more but not all disease replicates [14]. However, when applying existing methods (e.g., edgeR, DESeq and baySeq) and controlling FDR at 0.05, few true DE genes could be detected; furthermore, none of these four genes were reported to be DE genes. This is because the aforementioned methods assume equal dispersion across groups and only detect group mean difference. There is little power in testing the mean when only some disease replicates express differently from the normal group.

For special groups of diseases such as syndromes, which are characterized by significant various combinations of gene expression aberrations, some DE genes are shared by all disease replicates; others may be unique in one or more disease replicates, but not all. In the literature, no statistical methods have been developed to handle such cases where each disease replicate has a considerably different combination of DE genes. Existing methods were built for simple cases in which DE genes are shared by all disease replicates. When there is greater heteroscedasticity among disease replicates as shown in Fig. 1, one simple approach to improve power for DE detection is to test the mean and dispersion change simultaneously. The Voom method [16, 17], incorporated in the LIMMA package [18], attempted to model heteroscedasticity at the observation- and sample-specific levels by modeling the variance to be dependent on the mean and adding sample-specific weights based on sample quality. However, this approach would not achieve our objective because the Voom method adjusted all genes in a sample with one sample weight. For syndromes, each sample has a different combination of genes regardless of sample quality. In addition, the Voom method models the variance as a linear function of the mean, which is also not feasible in our scenario. Depending on the disease samples collected in an experiment, a varying group of DE genes can show differential expression in any number of disease samples. Thus, the mean-variance relationship cannot be established across DE genes and/or across experiments.

Throughout this paper, we use “DE genes” to refer to those genes whose mean expression levels change significantly in one or more (or all) replicates in the disease group compared to the normal group. Hence, equally expressed (EE) genes in our case refer to those whose mean expression levels are the same across all replicates in two comparison groups. We developed a statistical method, DESyn, which is short for differential expression analysis for syndromes, to test the mean and dispersion simultaneously. Due to the low number of replicates often used in NGS experiments, we adopted an empirical Bayes method to borrow information across genes to improve dispersion estimation for each gene and treatment group combination. We then designed a pooled permutation test to identify significant DE genes. In addition, confidence intervals based on NB distributions were used to further detect, for each DE gene, which replicate(s) in the disease group differed from the normal group. Next, for each afflicted replicate, we could find the unique combination of genes underlying disease along with commonly shared DE genes among all afflicted replicates. We illustrated our algorithm through its application to kidney tissue in the LOS study along with simulation studies that mimicked the real datasets. The R function used to conduct the study is available to download on github at https://github.com/cmrf7/DESyn.

## Method

The objective of our analysis is to develop a statistical method for syndromes that is more powerful than existing methods of DE gene detection between normal and disease groups. We assume normalized read counts follow NB distributions, thereby relaxing the restriction in the Poisson distribution requiring the mean and variance to be equal. Let *i* index treatment groups where *i*=1 denotes the normal group and *i*=2 denotes the disease group. Let *n*_*i*_ denote the number of replicates in group *i*. Let *Y*_*gij*_ denote the normalized read count for gene *g* (*g*=1,…,*G*) of treatment *i* (*i*=1,2) and replicate *j* (*j*=1,…,*n*_*i*_). Then, we have *Y*_*gij*_∼*iid*NB(*μ*_*gi*_,*ϕ*_*gi*_). Because NGS is a relatively expensive technology, *n*_*i*_’s are often small (e.g., three or four); thus, estimation of the dispersion parameter *ϕ*_*gi*_ is unreliable. edgeR, DESeq, and baySeq methods have attempted to improve these estimates by assuming equal dispersion across treatment groups; that is, *ϕ*_*g*1_=*ϕ*_*g*2_=*ϕ*_*g*_ for each gene *g*. They hoped that by assuming a common dispersion, data from two treatment groups could be pooled to better estimate dispersion. This assumption may be appropriate for simple cases, but for syndromes, ignoring extra dispersion in the disease group will limit the power in finding DE genes (see Fig. 1). An NB likelihood ratio test (LRT) shown in (1) can simultaneously examine the change in mean and dispersion. Specifically, for gene *g*, to test *H*_0_: *μ*_*g*1_=*μ*_*g*2_=*μ*_*g*_ and *ϕ*_*g*1_=*ϕ*_*g*2_=*ϕ*_*g*_, an LRT statistic is

1 $$ LR_{g}=-2\log\frac{sup\left\{L\left(\hat{\mu}_{g}, \hat{\phi}_{g}; \mathbf{y}_{g}\right)\right\}}{sup\left\{L\left(\hat{\mu}_{g1}, \hat{\phi}_{g1}, \hat{\mu}_{g2}, \hat{\phi}_{g2}; \mathbf{y}_{g}\right)\right\}},  $$

where **y**_*g*_ is the vector of observations for gene *g*. $\hat {\mu }_{g}$ and $\hat {\phi }_{g}$ are maximum likelihood estimates (MLEs) when assuming the same mean and dispersion across two treatment groups. $\hat {\mu }_{g1}$, $\hat {\phi }_{g1}$, $\hat {\mu }_{g2}$, and $\hat {\phi }_{g2}$ are MLEs, respectively, for the normal and disease groups. When sample size is large, *LR*_*g*_ approximately follows a *χ*^2^ distribution with two degrees of freedom. When *LR*_*g*_ is large, such that $sup\left \{L\left (\hat {\mu }_{g1}, \hat {\phi }_{g1}, \hat {\mu }_{g2}, \hat {\phi }_{g2}; \mathbf {y}_{g}\right)\right \}$ is significantly larger than $sup\left \{L\left (\hat {\mu }_{g}, \hat {\phi }_{g}; \mathbf {y}_{g}\right)\right \}$, we can reject *H*_0_ and claim that either the mean or dispersion differ between the two comparison groups for gene *g*.

Due to the low number of replicates in each treatment group, MLEs are not robust, especially for dispersion parameters. To obtain stable dispersion estimates, we adopt the idea in edgeR of assigning a common hyper prior on the dispersion parameters of all genes to share information across genes. Specifically, in (1), we assume $\hat {\phi }_{g} | \phi _{g} \sim \mathrm {N}\left (\phi _{g}, \tau ^{2}_{g}\right)$ and $\phi _{g}\sim \mathrm {N}\left (\phi _{0}, \tau ^{2}_{0}\right)$. Similarly, we also assume $\hat {\phi }_{g1} | \phi _{g1} \sim \mathrm {N}(\phi _{g1}, \tau ^{2}_{g1})$, $\phi _{g1}\sim \mathrm {N}\left (\phi _{1}, \tau ^{2}_{1}\right)$ and $\hat {\phi }_{g2} | \phi _{g2} \sim \mathrm {N}\left (\phi _{g2}, \tau ^{2}_{g2}\right)$, $\phi _{g2}\sim \mathrm {N}\left (\phi _{2}, \tau ^{2}_{2}\right)$. By using the same inference procedure in edgeR [4], the Bayes posterior mean estimators are $\hat {\phi }^{B}_{g}=\mathrm {E}\left (\phi _{g}|\hat {\phi }_{g}\right)=\left (\hat {\phi }_{g}/\tau ^{2}_{g}+\phi _{0}/\tau ^{2}_{0}\right)/\left (1/\tau ^{2}_{g}+1/\tau ^{2}_{0}\right)$, $\hat {\phi }^{B}_{g1}=\mathrm {E}\left (\phi _{g1}|\hat {\phi }_{g1}\right)=\left (\hat {\phi }_{g1}/\tau ^{2}_{g1}+\phi _{1}/\tau ^{2}_{1}\right)/\left (1/\tau ^{2}_{g1}+1/\tau ^{2}_{1}\right)$ and $\hat {\phi }^{B}_{g2}=\mathrm {E}\left (\phi _{g2}|\hat {\phi }_{g2}\right)=\left (\hat {\phi }_{g2}/\tau ^{2}_{g2}+\phi _{2}/\tau ^{2}_{2}\right)/\left (1/\tau ^{2}_{g2}+1/\tau ^{2}_{2}\right)$. $\hat {\phi }^{B}_{g}$, $\hat {\phi }^{B}_{g1}$, and $\hat {\phi }^{B}_{g2}$ are considered improved estimates of $\hat {\phi }_{g}$, $\hat {\phi }_{g1}$, and $\hat {\phi }_{g2}$. The hyper priors *ϕ*_0_, $\tau ^{2}_{0}$, *ϕ*_1_, $\tau ^{2}_{1}$, and *ϕ*_2_, $\tau ^{2}_{2}$ are estimated using observations from all genes via the same inference procedure described in edgeR. Because there are tens of thousands of genes in a whole genome, the estimates of hyper prior parameters are accurate, and the dispersion parameter estimates from sharing information across genes are robust.

By replacing $\hat {\phi }_{g}$, $\hat {\phi }_{g1}$, and $\hat {\phi }_{g2}$ in (1) by $\hat {\phi }^{B}_{g}$, $\hat {\phi }^{B}_{g1}$, and $\hat {\phi }^{B}_{g2}$, we obtain an updated test statistic in (2).

2 $$ LR^{B}_{g}=-2\log\frac{sup\left\{L\left(\hat{\mu}_{g}, \hat{\phi}^{B}_{g}; \mathbf{y}_{g}\right)\right\}}{sup\left\{L\left(\hat{\mu}_{g1},\hat{\phi}^{B}_{g1}, \hat{\mu}_{g2}, \hat{\phi}^{B}_{g2}; \mathbf{y}_{g}\right)\right\}}.  $$

Notice that $LR^{B}_{g}$ no longer follows a *χ*^2^ distribution, and it is difficult to analytically derive its null distribution. We adopt the idea from [19] and designed a pooled permutation method to estimate its null distribution. Specifically, we follow these steps:

1. For each gene *g*, calculate the *p*-value, *p*_*g*_, based on the NB LRT in (1).
2. Collect all genes whose *p*_*g*_≥0.1 and call them the set of null-like genes.
3. For the set of null-like genes only, permute the treatment group among the *n*=*n*_1_+*n*_2_ replicates. Suppose the total number of possible permutations is *M*. For each permutation *m* (*m*=1,…,*M*), compute $LR^{B(m)}_{g}$ in (2). The empirical Bayes estimates of the parameters and hyper parameters are estimated in each permutation using all genes (i.e., null-like and non-null-like genes). Then, the empirical distribution of the set $\left \{LR^{B(m)}_{g}: m=1, \dots, M, \text { and {g} is a null-like gene}.\right \}$ estimates the null distribution of $LR^{B}_{g}$.
4. Compute the estimated *p*-value for each gene *g*, $p^{B}_{g}$, using $LR^{B}_{g}$ and the estimated null distribution of $LR^{B}_{g}$ in Step 3.

The choice of using 0.1 as the cutoff in step 2 follows the recommendation from [19], which presents studies on the choice of a proper default cutoff. Finally, we use Storey’s method [20] to control FDR at the desired level.

For microarray data analysis, [21, 22] argued that borrowing information across all genes might lead to over-correction. A better approach is to apply gene-specific or group-specific prior based on historic data to share information only across genes with similar variances. These ideas can be adapted for sequencing data analysis as alternative approaches to overcome the problem of having low number of replicates.

## Results and discussion

In this section, we demonstrate the performance of our proposed method using real biological experiment data and simulated datasets. In addition, we compare our method DESyn to the popular LIMMA and edgeR approaches. Notice that these methods are designed for experiments where all disease samples share the same set of DE genes, whereas our approach is intended for syndromes where each disease sample has a different set of DE genes.

### Large offspring syndrome gene expression data

To illustrate our method’s performance, we used kidney tissue data from the LOS study in [14, 15]. The raw FASTQ files are publicly available at Gene Expression Omnibus with accession no. GSE63509. LOS is an overgrowth phenotype observed in ruminant fetuses, which mimics the human fetal overgrowth condition Beckwith-Wiedemann syndrome (BWS). BWS is the most common congenital overgrowth disorder and has an estimated worldwide frequency of 1 in 13,700 live births [23, 24]. Some commonly observed features in BWS patients are macroglossia, neonatal and postnatal macrosomia, hemihypertrophy, ear malformations, and abdominal wall defects [25–27]. In [14, 15]’s study, they used cows as study animals to identify genes related to LOS syndrome. The sequencing experiment contained four control samples and four LOS female samples, respectively. After discarding genes with sum counts no greater than 10 across two treatment groups, 19,946 genes remained to be tested. We then used the trimmed mean of M-values (TMM) method [28] to normalize the raw data. To detect DE genes in this LOS study, we used three approaches: the LIMMA method, edgeR method, and our proposed method DESyn. All three methods assume an NB distribution. The LIMMA and edgeR methods only test the mean difference, whereas our proposed DESyn method can test change in both mean and dispersion while improving power by estimating dispersion parameters more accurately.

By controlling FDR at 0.05, the LIMMA method reported 13 DE genes; the edgeR method reported 55 DE genes; and the DESyn method reported 2716 DE genes across all four LOS samples. Among the 13 declared DE genes by LIMMA, 11 were declared by DESyn; among 55 declared DE genes by edgeR, 38 were declared by DESyn. In addition, 9 genes were detected by both LIMMA and edgeR, all of which were detected by DESyn. The four example genes in Fig. 1 were all identified as DE genes by the DESyn method, but none were reported to be DE genes by LIMMA or edgeR methods. Genotyping results confirmed that the four example genes were all monoallelically expressed in controls, whereas one or more replicates were biallelically expressed in LOS fetuses. For instance, gene NNAT exhibited monoallelic expression from the paternal allele for the control group and the first two replicates in the LOS group. For the third and fourth replicates in the LOS group, NNAT showed loss of imprinting and exhibited biallelic expression from the paternal and maternal alleles. Per the GeneCard database (www.genecards.org), NNAT is associated with tumor growth (*p*-value =1.0×10^−14^), and PEG3, PLAGL1, and SNRPN are all associated with body size growth (*p*-value =1.0×10^−16^) [14].

**Fig. 2:**
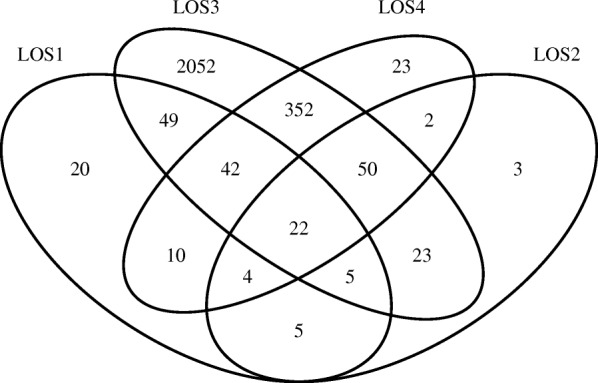
Venn diagram of discovered DE genes. Venn diagram for unique and shared DE genes among four LOS replicates (FDR=0.05). LOS1, LOS2, LOS3, LOS4 are the 4 replicates in the LOS group

Among the DE genes detected by our method, the following warranted further consideration: which DE genes were commonly shared by all LOS samples, which were shared by some LOS samples, and which were unique to one LOS sample. To identify DE genes accordingly, for each detected DE gene by DESyn, we computed the confidence interval for the normal group with the estimated mean $\hat {\mu }_{g1}$ and dispersion parameter $\hat {\phi }^{B}_{g1}$. We then compared each of the four LOS observations with the estimated confidence interval of the normal group mean. We applied a Bonferroni adjustment to control the family-wise error rate (FWER) at level 0.05. By this method, we are able to identify which combination of these DE genes led to LOS occurrence in each of the four LOS samples. Results for the four LOS replicates are summarized in Fig. 2. In particular, 22 detected DE genes were shared by all four LOS samples.

Previous research study [14] pointed out a presumably positive correlation between the number of DE genes due to loss of imprinting in each LOS fetus and fetuses’ body weights. The four LOS sample body weights were 514 g, 518 g, 620 g, and 714 g, respectively. Using the DESyn method, we found 157 DE genes for LOS sample No. 1, 114 DE genes for sample No. 2, 2595 DE genes for sample No. 3, and 505 DE genes for sample No. 4. The numbers of DE genes detected for these four LOS samples exhibited a weak positive linear relationship with the sample body weights (correlation coefficient *ρ*=0.34).

### Simulation studies

In our simulation studies, we compared our proposed DESyn method with the LIMMA and edgeR methods. We simulated different settings and compared these approaches accordingly. In each simulation study, we simulated data for 5000 genes in the control and disease groups.

#### Simulation design

Simulation studies based on real datasets best demonstrate a method’s practical utility. We generated data for the normal group from NB distributions, where means and dispersions were estimated from 5000 randomly selected genes from the normal group in the LOS study. For the data in the disease group, EE genes were sampled from the NB distributions with the same parameters as the normal group in the real dataset. To generate data for DE genes in the disease group, we considered the following three scenarios:

- Scenario 1: No mean difference, but the disease group has a different dispersion compared to the normal group;
- Scenario 2: No mean difference, but some replicate(s) in the disease group have a different dispersion and other replicate(s) have the same dispersion compared to the normal group;
- Scenario 3: Both mean and dispersion differences exist between the normal and disease groups.

In simulation study 1, for the disease group, we simulated 4000 EE genes and 1000 DE genes from Scenario 1. Specifically, we defined scale parameter *δ*_*ϕ*_∼Beta(*α*=2,*β*=2) and size parameter *X*_*ϕ*_=0.4. For each of the 1000 DE genes in the disease group, we randomly simulated *δ*_*ϕ*_ and let *ϕ*_*g*2_=*ϕ*_*g*1_+(*X*_*ϕ*_×*δ*_*ϕ*_). The size parameter was set at a value to ensure the simulated DE genes would be neither too easy nor too difficult to detect. We chose *α* and *β* parameter values for *δ*_*ϕ*_ to generate an equal number of DE genes with small and large dispersion, with most demonstrating a median dispersion difference between the two groups. We also set *μ*_*g*1_=*μ*_*g*2_ for all replicates.

In simulation study 2, for the disease group, we simulated 4000 EE genes and 1000 DE genes from Scenario 2. For the *g*th gene of the DE genes in the disease group, we first simulated *k*_*g*_ from discrete uniform distribution U{1,2,3,4}. Then, for the last *k*_*g*_ replicates of the *g*th DE gene in the disease group, we let *ϕ*_*g*2_=*ϕ*_*g*1_+(*X*_*ϕ*_×*δ*_*ϕ*_). Parameters *X*_*ϕ*_ and *δ*_*ϕ*_ were simulated similarly to simulation study 1. For other replicates of the *g*th gene of the DE genes in the disease group (i.e., replicates 1 to (*k*_*g*_−1)), we kept *ϕ*_*g*2_=*ϕ*_*g*1_. We set *μ*_*g*1_=*μ*_*g*2_ for all replicates.

In simulation study 3, for the disease group, we simulated 4000 EE genes and 1000 DE genes from Scenario 3. We defined scale parameter *δ*_*μ*_∼Beta(*α*=2,*β*=4) and size parameter *X*_*μ*_=2. For each of the 1000 DE genes in the disease group, we let *ϕ*_*g*2_=*ϕ*_*g*1_+(*X*_*ϕ*_×*δ*_*ϕ*_) where *δ*_*ϕ*_∼Beta(*α*=2,*β*=2) and *X*_*ϕ*_=0.3, and *μ*_*g*2_=*μ*_*g*1_+(*X*_*μ*_×*δ*_*μ*_×*σ*_*g*1_) where *σ*_*g*1_ is the standard deviation of the NB distribution for the normal group. The *α* and *β* parameters in the distribution of *δ*_*μ*_ were chosen so that most gene expression mean differences were small and a few were large, as is often the case for real gene expression datasets.

Finally, in simulation study 4, we simulated 3950 EE genes, 350 DE genes from Scenario 1, 350 DE genes from Scenario 2, and 350 DE genes from Scenario 3, where the simulation methods for each scenario were identical to those in simulation studies 1, 2, and 3.

For each of these four simulation studies, we considered four, five, and six replicates in the normal and disease groups, respectively.

#### Simulation results

We repeated each of the four simulation settings 50 times. The following results are based on the repetitions’ average. We also reported standard deviation (SD) for the summary statistics based on the 50 repeated simulations.

**Fig. 3:**
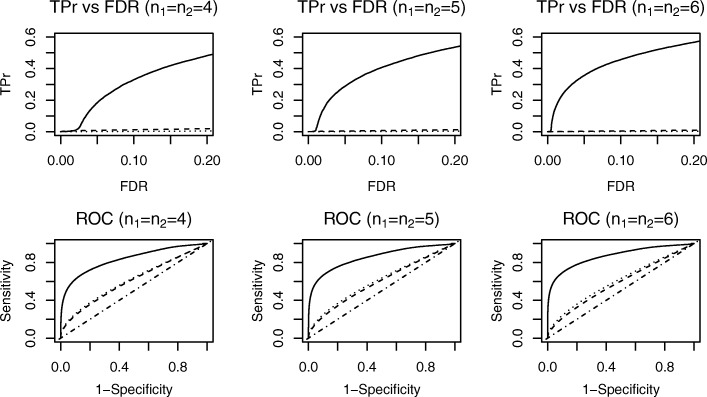
Simulation Study 1. Dotted curves stand for LIMMA method; Dashed curves stand for edgeR method; Solid curves stand for our proposed method; Dot-dashed line shows the diagonal line. Top row shows TPr comparison at fixed levels of FDR. Bottom row shows comparison of ROC curves

Figure 3 shows the comparison results for simulation study 1. Specifically, the three figures in the first row demonstrate a true positive rate (TPr) when controlling FDR at a fixed level for replicates *n*_1_=*n*_2_= 4, 5, and 6, respectively. Our DESyn method, compared to the LIMMA and edgeR approaches, exhibited substantially greater power in detecting DE genes. In simulation study 1, DE genes had the same means between normal and disease groups and differed only in dispersion parameters. Thus, the LIMMA and edgeR methods, which only tested the mean difference, did not have any power. When there were more replicates in each treatment group, the power of the DESyn method improved with a fixed FDR level. However, the power of the LIMMA and edgeR approaches did not improve as the number of replicates increased, likely because these methods cannot detect dispersion differences regardless of how many replicates are available. The second row of Fig. 3 shows ROC curves with three different numbers of replicates. Our proposed method had the best performance among the selected methods. With more replicates, the ROC curves improved for our approach. We also summarized results in Table 1. Specifically, by controlling FDR at 0.05, we reported the number of true positives and the number of total positives averaged across 50 simulations. Our proposed method reported a significantly higher number of DE genes and true DE genes compared to the other two methods. In addition, we also calculated the actual FDR and its SD across 50 simulations. Statistics indicate that the actual FDR of our proposed method decreases as the number of replicates increases. The last three columns in the Table 1 display the area under the ROC curves (AUCs), demonstrating that the DESyn method has the largest AUCs of the three methods and thus ranks genes better than the other two competing methods. Although we directly adopted the recommendation from [19] to use 0.1 as the cutoff to select null-like genes in the pooled permutation test, we also compared using 0.1 versus 0.2 as the cutoff. We found that across simulations, using 0.1 as the cutoff has a slightly higher average TPr than using 0.2 when FDR is controlled at 0.05.

**Table 1:** Simulation results for simulation studies 1, 2, 3, and 4 with number of replicates 4, 5, and 6, respectively

Study	Rep	True positives			Actual FDR			AUC		
		(Total positives)			(SD)			(SD)		
		Limma	edgeR	Proposed	Limma	edgeR	Proposed	Limma	edgeR	Proposed
1	4	1.34	10.32	194.92	0.0000	0.0518	0.0484	0.6141	0.6038	0.8332
		(1.34)	(10.92)	(207.26)	(0.0000)	(0.0619)	(0.0287)	(0.0117)	(0.0102)	(0.0093)
	5	1.04	6.32	294.38	0.0100	0.0291	0.0409	0.6105	0.5946	0.8541
		(1.06)	(6.60)	(307.54)	(0.0707)	(0.0591)	(0.0155)	(0.0122)	(0.0117)	(0.0083)
	6	0.96	4.62	361.04	0.0000	0.0445	0.0420	0.6053	0.5839	0.8676
		(0.96)	(4.80)	(377.26)	(0.0000)	(0.1230)	(0.0138)	(0.0107)	(0.0097)	(0.0075)
2	4	0.60	2.82	26.44	0.0000	0.0632	0.0192	0.5771	0.5925	0.7554
		(0.60)	(3.08)	(27.46)	(0.0000)	(0.1295)	(0.0310)	(0.0122)	(0.0094)	(0.0112)
	5	0.50	1.76	86.90	0.0200	0.0857	0.0313	0.5675	0.5804	0.7698
		(0.52)	(2.04)	(89.88)	(0.1414)	(0.2062)	(0.0220)	(0.0109)	(0.0100)	(0.0076)
	6	0.16	1.08	136.00	0.0000	0.0367	0.0265	0.5617	0.5721	0.7792
		(0.16)	(1.14)	(139.78)	(0.0000)	(0.1625)	(0.0155)	(0.0105)	(0.0105)	(0.0093)
3	4	1.10	8.94	129.22	0.0067	0.0421	0.0373	0.6277	0.6474	0.8359
		(1.12)	(9.32)	(137.30)	(0.0471)	(0.0743)	(0.0322)	(0.0107)	(0.0122)	(0.0072)
	5	0.68	9.02	239.14	0.0000	0.0346	0.0393	0.6176	0.6413	0.8551
		(0.68)	(9.32)	(249.58)	(0.0000)	(0.0817)	(0.0161)	(0.0110)	(0.0122)	(0.0098)
	6	0.66	6.94	331.44	0.0000	0.0381	0.0408	0.6158	0.6446	0.8731
		(0.66)	(7.14)	(345.90)	(0.0000)	(0.1041)	(0.0136)	(0.0096)	(0.0095)	(0.0061)
4	4	0.72	7.68	121.68	0.0000	0.0554	0.0378	0.6083	0.6101	0.8069
		(0.72)	(8.12)	(128.50)	(0.0000)	(0.0913)	(0.0293)	(0.0106)	(0.0122)	(0.0105)
	5	0.94	5.86	211.54	0.0000	0.0326	0.0346	0.6035	0.6036	0.8257
		(0.94)	(6.12)	(219.70)	(0.0000)	(0.0711)	(0.0166)	(0.0094)	(0.0107)	(0.0080)
	6	0.66	4.06	296.70	0.0200	0.0290	0.0356	0.5966	0.5976	0.8410
		(0.68)	(4.20)	(307.96)	(0.1414)	(0.1022)	(0.0132)	(0.0104)	(0.0101)	(0.0074)

Summary statistics including number of true positives with total number of positives in parentheses, the actual FDR by controlling FDR at 0.05 level and its standard error in parentheses, and the area under ROC curve with standard error in parentheses

**Fig. 4:**
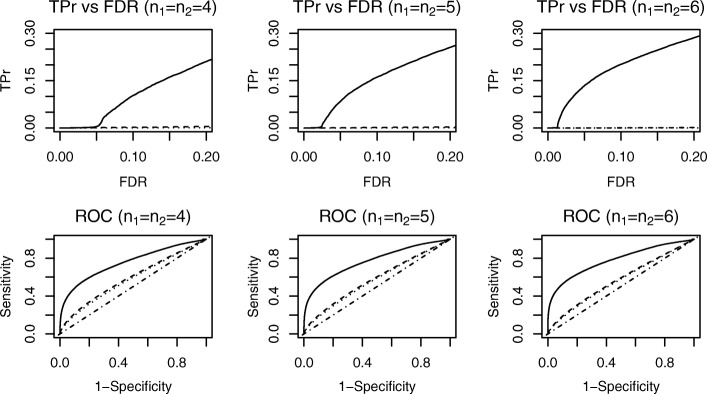
Simulation Study 2. Dotted curves stand for LIMMA method; Dashed curves stand for edgeR method; Solid curves stand for our proposed method; Dot-dashed line shows the diagonal line. Top row shows TPr comparison at fixed levels of FDR. Bottom row shows comparison of ROC curves

Similarly, Fig. 4 shows the results from simulation study 2, in which only part of the disease samples had different dispersions than the normal samples. In simulation study 1, however, all disease samples had different dispersions than the normal group; thus, simulation study 2 presented a more challenging scenario. Results are depicted in Fig. 4 and Table 1: with a fixed level of FDR at 0.05, all three methods had reduced power compared to simulation study 1. However, the DESyn method still exhibited greater power than the other two methods for DE gene detection. Like simulation study 1, neither the LIMMA nor the edgeR method showed improved power as the number of replicates increased, whereas our proposed method improved its power significantly. Our approach also outperformed the LIMMA and edgeR methods with respect to the ranking of genes reflected in the ROC curves. These results were expected because the LIMMA and edgeR methods only detected the mean difference while assuming equal dispersion across treatment groups.

**Fig. 5:**
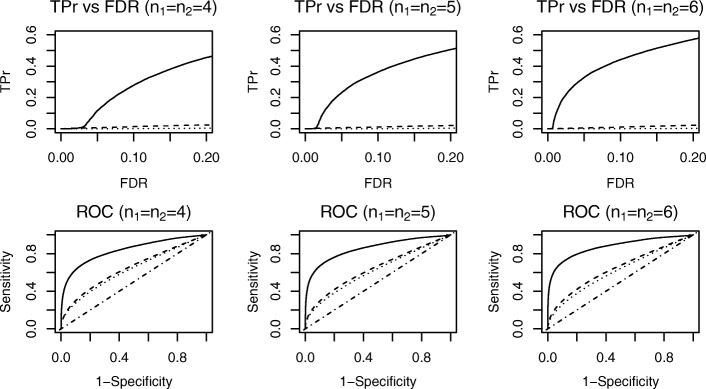
Simulation Study 3. Dotted curves stand for LIMMA method; dashed curves stand for edgeR method; solid curves stand for our proposed method; Dot-dashed line shows the diagonal line. Top row shows TPr comparison at fixed levels of FDR. Bottom row shows comparison of ROC curves

Figure 5 shows the results of simulation study 3, where DE genes differed in both mean and dispersion. Similar to the results of simulation study 1, the DESyn method had a substantially greater power than the LIMMA and edgeR methods in DE gene detection, as evidenced by the number of true positives. Our method was also superior to the competing methods in gene ranking, demonstrated by the ROC curves and AUCs in Fig. 5 and Table 1.

**Fig. 6:**
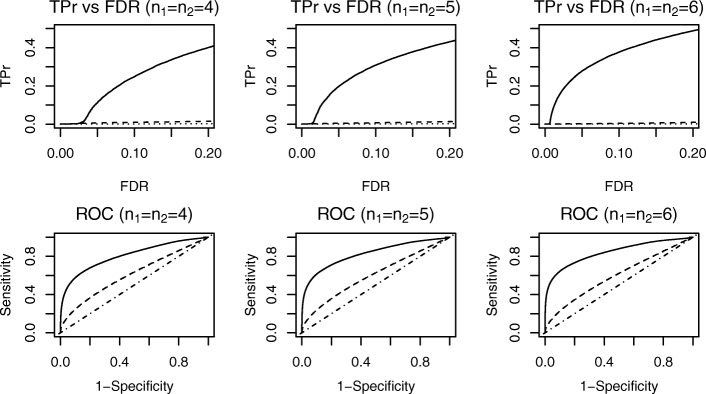
Simulation Study 4. Dotted curves stand for LIMMA method; dashed curves stand for edgeR method; solid curves stand for our proposed method; Dot-dashed line shows the diagonal line. Top row shows TPr comparison at fixed levels of FDR. Bottom row shows comparison of ROC curves

In simulation study 4, the DE genes combined the three scenarios in simulation studies 1, 2, and 3. Results appear in Fig. 6 and Table 1. The DESyn method performed better than the LIMMA and edgeR approaches with respect to the power in DE gene detection and DE gene ranking.

It is possible that one outlier replicate in the disease group can be detected as a signal of a DE gene when testing the change in mean and dispersion simultaneously using our proposed method. The same is true when testing the mean only. Outliers with a large deviation will result in false positives whether we test the mean alone or mean and dispersion simultaneously. In our DE gene detection scenario, we considered syndromes where extant literature has shown that the syndrome is characterized by each afflicted individual having a different combination of DE genes. In this case, traditional gene expression analysis methods have no power of DE gene detection when a DE gene is only shared by some disease replicates.

## Conclusions

In this study, we proposed an empirical Bayes statistic to identify DE genes by accounting for change in the mean and dispersion when comparing normal and disease groups. Our motivation came from real data analysis regarding LOS syndrome, where different combinations of DE genes lead to the same disease. Based on the empirical Bayes statistic, we further developed a pooled permutation method for statistical inferences. We analyzed the real dataset of kidney tissue in the LOS study. Of the detected DE genes, several were biologically verified in the literature. We further utilized a parametric method based on NB distributions and Bayes estimates to find commonly shared DE genes in all LOS fetus samples and DE genes only shared by some LOS samples. These results could not be obtained by existing methods. Moreover, we conducted simulation studies based on the real dataset from the LOS study. Under different settings, we proved the benefits and advantages of our proposed method.

## Abbreviations

AUC: Area under ROC curve
BWS: Beckwith-Wiedemann syndrome
DE: Differentially expressed
EE: Equally expressed
FDR: False discovery rate
FWER: Family wise error rate
LOS: Large offspring syndrome
LRT: Likelihood ratio test
MLE: Maximum likelihood estimate
NB: Negative binomial
NGS: Next-generation sequencing
SD: Standard deviation
TMM: Trimmed mean of M-values
TPr: True positive rate
TSPM: Two-stage Poisson model
